# A Comprehensive Strategy to Evaluate Compatible Stability of Chinese Medicine Injection and Infusion Solutions Based on Chemical Analysis and Bioactivity Assay

**DOI:** 10.3389/fphar.2017.00833

**Published:** 2017-11-15

**Authors:** Jian-Ping Li, Yang Liu, Jian-Ming Guo, Er-Xin Shang, Zhen-Hua Zhu, Kevin Y. Zhu, Yu-Ping Tang, Bu-Chang Zhao, Zhi-Shu Tang, Jin-Ao Duan

**Affiliations:** ^1^Jiangsu Collaborative Innovation Center of Chinese Medicinal Resources Industrialization, Nanjing University of Chinese Medicine, Nanjing, China; ^2^Jiangsu Key Laboratory for High Technology Research of TCM Formulae, Nanjing University of Chinese Medicine, Nanjing, China; ^3^Buchang Pharmaceutical Co., Ltd., Xi’an, China; ^4^Shaanxi University of Chinese Medicine, Xianyang, China

**Keywords:** Danhong injection, compatibility, stability, infusion solution, Chinese medicine injection

## Abstract

Stability of traditional Chinese medicine injection (TCMI) is an important issue related with its clinical application. TCMI is composed of multi-components, therefore, when evaluating TCMI stability, several marker compounds cannot represent global components or biological activities of TCMI. Till now, when evaluating TCMI stability, method involving the global components or biological activities has not been reported. In this paper, we established a comprehensive strategy composed of three different methods to evaluate the chemical and biological stability of a typical TCMI, Danhong injection (DHI). UHPLC-TQ/MS was used to analyze the stability of marker compounds (SaA, SaB, RA, DSS, PA, CA, and SG) in DHI, UHPLC-QTOF/MS was used to analyze the stability of global components (MW 80–1000 Da) in DHI, and cell based antioxidant capability assay was used to evaluate the bioactivity of DHI. We applied this strategy to assess the compatible stability of DHI and six infusion solutions (GS, NS, GNS, FI, XI, and DGI), which were commonly used in combination with DHI in clinic. GS was the best infusion solution for DHI, and DGI was the worst one based on marker compounds analysis. Based on global components analysis, XI and DGI were the worst infusion solutions for DHI. And based on bioactivity assay, GS was the best infusion solution for DHI, and XI was the worst one. In conclusion, as evaluated by the established comprehensive strategy, GS was the best infusion solution, however, XI and DGI were the worst infusion solutions for DHI. In the compatibility of DHI and XI or DGI, salvianolic acids in DHI would be degraded, resulting in the reduction of original composition and generation of new components, and leading to the changes of biological activities. This is the essence of instability compatibility of DHI and some infusion solutions. Our study provided references for choosing the reasonable infusion solutions for DHI, which could contribute the improvement of safety and efficacy of DHI. Moreover, the established strategy may be applied for the compatible stability evaluation of other TCMIs.

## Introduction

Traditional Chinese medicine injection (TCMI) is an innovative formulation with high bioavailability and good efficacy, and TCMI is widely used to treat acute and severe diseases in China. Stability and safety of TCMI are important issues related with its clinical application, instability and unsafety of TCMI are often caused by improper combination with other medicines. Thus, TCMIs are strictly prohibited to be mixed with each other or with chemical medicine in clinic. However, most of TCMIs are concentrated liquid formulation, which must be mixed with infusion solutions to obtain proper concentrations before they are injected to patients, the same procedure as chemical medicine injections. Therefore, evaluation of the compatibility of TCMI and infusion solutions is necessary and important for TCMI to be used in an appropriate way.

The importance of compatible stability of chemical medicine injections and infusion solutions have been reported in many studies (Binnor et al., 2013; Chen et al., 2015a,b; Myers et al., 2016). As chemical medicine injections generally contain only single active component, the change of single active component can represent the change of injection stability. Therefore, using single active component as marker to evaluate compatible stability is suitable for chemical medicine injections. While for TCMI, the chemical components in TCMI are quite complicated, which make it much more difficult to choose appropriate markers to evaluate the stability of TCMI. Currently, evaluation of compatible stability of TCMI and infusion solutions are still based on single or several marker compounds, while the changes of global components, or biological activity are rarely evaluated (Li J. et al., 2014).

Danhong injection (DHI) is a mixed extraction of two typical Chinese species, the radix and rhizome of *Salvia miltiorrhiza* Bunge (Labiatae) and the dry flower of *Carthamus tinctorius* L. (Asteraceae). In China, DHI is widely applied for the treatments of cardiovascular and cerebrovascular diseases, such as myocardial infarction, cerebral thrombosis, and ischemic encephalopathy. The efficacy of DHI on vascular repair after percutaneous coronary intervention has been approved by a recent randomized clinical trial (Hu et al., 2016). In China, the annual sale of DHI is 800 million dollars in 2016, which has made DHI one of the most promising drugs to be “blockbuster.”

According to the clinical operating instructions, DHI should be diluted with 5% glucose injection (GS) before use, while for patients with diabetes and other related diseases, DHI should be diluted with 0.9% sodium chloride injection (NS) instead. However, 5% glucose – 0.9% sodium chloride injection (GNS), 10% fructose injection (FI), 5% xylitol injection (XI) and 6% dextran 40 – 5% glucose injection (DGI) are also used as infusion solutions for DHI in clinic. Currently, no systematic studies have been reported to evaluate the compatible stability of DHI and these infusion solutions. Evaluation of DHI-GS or DHI-NS compatible stability have been reported, while the compatible stability of DHI and other infusion solutions are less involved. Furthermore, when evaluating the compatible stability of DHI-GS or DHI-NS, only physical-chemical properties such as insoluble particles and pH values are involved, changes of chemical components or pharmacological activities are often ignored (Li J. et al., 2014).

**FIGURE 1:**
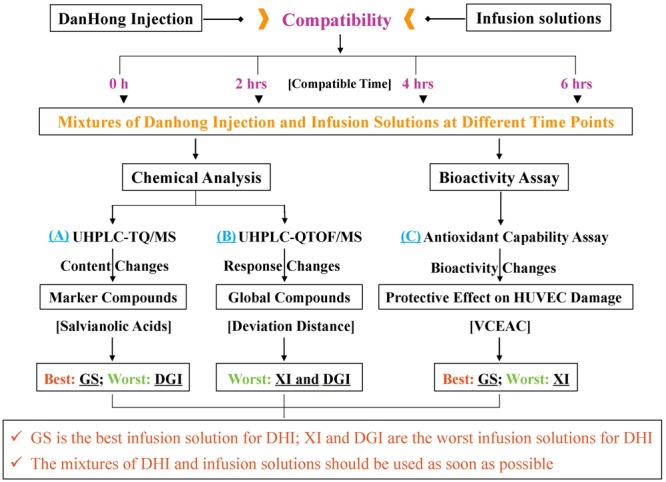
A comprehensive strategy to evaluate compatible stability of Danhong injection (DHI) and infusion solutions by chemical analysis and bioactivity assay. GS is the best infusion solution for DHI; XI and DGI are the worst infusion solutions for DHI.

Multicomponent is the characteristic of TCMI, therefore, when evaluating TCMI stability, several marker compounds in TCMI are not enough to represent global components or biological activities of TCMI. In this paper, we developed a comprehensive strategy composed of three different methods to evaluate the compatible stability of DHI and infusion solutions, thus we could propose the best and worst infusion solutions for DHI based on a more comprehensive analysis (**Figure 1**). Moreover, the strategy reported in our study may be applied for the compatible stability evaluation of other TCMIs.

## Materials and Methods

### Materials

**FIGURE 2:**
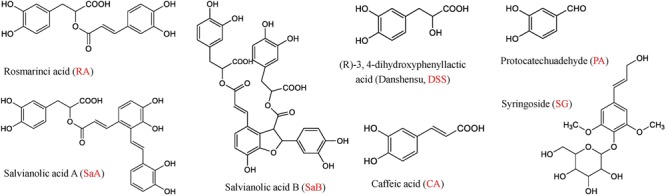
Chemical structures of rosmarinic acid (RA), salvianolic acid A (SaA), salvianolic acid B (SaB), (R)-3,4-dihydroxyphenyllactic acid (Danshensu, DSS), protocatechuic aldehyde (PA), caffeic acid (CA) and syringoside (SG).

DHI was provided by Buchang Pharmaceutical Co., Ltd. (Shandong, China) (Lot Number: 15081038). GS, NS, GNS, FI, XI, and DGI were all purchased from Jiangsu Province Hospital of Traditional Chinese Medicine (Nanjing, China). Salvianolic acid A (SaA), salvianolic acid B (SaB), rosmarinic acid (RA), (R)-3,4-dihydroxyphenyllactic acid sodium salt (danshensu, DSS), protocatechuic aldehyde (PA), caffeic acid (CA) and syringoside (SG) (**Figure 2**) were all purchased from Chinese materials research center (Nanjing, China).

Formic acid was purchased from Merck KGaA (Darmstadt, Germany). Ultra-pure water was purified by an EPED super purification system (Nanjing, China). HPLC-MS grade acetonitrile was purchased from TEDIA Company Inc. (Fairfield, United States). Other chemical reagents were all analytical grade and purchased from Sinopharm Chemical Reagent Co., Ltd. (Shanghai, China).

Human umbilical vein endothelial cells (HUVEC) were purchased from American Type Culture Collection (ATCC) (Manassas, VA, United States). F-12K nutrient mixture (1×), fetal bovine serum (FBS), penicillin-streptomycin (P/S) and 0.25% trypsin-EDTA were all purchased from Gibco (Grand Island, NY, United States). Vitamin C (L-ascorbic acid), Dimethyl sulfoxide (DMSO), 3-(4,5-dimethyl-2-thiazolyl)-2,5-diphenyl-2H-tetrazolium bromide (MTT) and 30% hydrogen peroxide (H_2_O_2_) were purchased from Sigma-Aldrich Co. LLC. (St. Louis, MO, United States). Heparin sodium salt (from porcine intestinal mucosa) was purchased from Alfa Aesar Chemicals Co., Ltd. (Shanghai, China). Endothelial cell growth supplement (ECGS) was purchased from ScienCell Research Laboratories (Carlsbad, CA, United States).

### Chemical Analysis of Marker Compounds by UHPLC-TQ/MS

Phenolic acids are major bioactive compounds in DHI and commonly used as marker components for quality control of DHI (Liu H.T. et al., 2013; Liu X. et al., 2013). Here, seven major phenolic acids including SaA, SaB, DSS, RA, PA, CA, and SG were chosen as marker compounds of DHI. We established a quantitative method of simultaneous determination of seven phenolic acids in DHI by UHPLC-TQ/MS, and evaluated the compatible stability according to the content changes of these marker compounds when DHI was mixed with six infusion solutions (GS, NS, GNS, FI, XI, and DGI) for different time (0, 2, 4, and 6 h) at room temperature (25°C).

#### Liquid Chromatography

Chromatographic analysis was performed on Acquity ultra high performance liquid chromatography (UHPLC) BEH C18 column (2.1 mm i.d. × 100 mm, 1.7 μm) using a Acquity UHPLC^TM^ system (Waters Corp., Milford, MA, United States). The column and auto-sampler were maintained at 35 and 20°C, respectively. The mobile phase, at a flow rate of 0.4 ml/min, consisted of (A) formic acid-water (1:1000, v/v) and (B) acetonitrile. The conditions of gradient eluting were optimized as the following: 14–21% B (0–1.0 min), 21–40% B (1.0–4.0 min), 40–95% B (4.0–4.2 min), 95–95% B (4.2–5.0 min), 95–14% B (5.0–5.2 min). The injection volume was 1 μl.

#### Mass Spectrometry

Mass spectrometry analysis was performed by using Xevo Triple Quadrupole Mass Spectrometer (TQ/MS) (Waters Corp., Milford, MA, United States) equipped with an electrospray ionization source (ESI). The quantification was performed by using selective reaction monitoring (SRM). The parameters for detection were set as the following: capillary voltage 3.0 kV; source temperature 150°C; desolvation temperature 550°C; cone gas flow 50 l/h; desolvation gas flow 1000 l/h. The optimal detection conditions for each phenolic acid were listed in **Table 1**.

**Table 1:** Optimal detection conditions for marker compounds by UPLC-TQ/MS.

Maker compounds	Ionization mode	Precursor ion (m/z)	Product ion (m/z)	Cone Voltage (kV)	Collision Energy (eV)	Retention Time (min)
SaA	ES^-^	493.16	295.09	28.0	18.0	3.99
SaB	ES^-^	717.29	519.17	26.0	18.0	3.63
RA	ES^-^	359.22	197.06	30.0	16.0	3.38
DSS	ES^+^	199.08	153.03	10.0	6.0	1.21
PA	ES^+^	138.83	111.07	16.0	12.0	1.93
CA	ES^+^	181.03	88.89	12.0	26.0	2.09
SG	ES^+^	395.22	232.19	34.0	28.0	1.50

#### Method Validation

Standard compounds were dissolved in 10% methanol to make standard stock solutions, respectively. The mixed stock solution was obtained by mixing seven standard stock solutions, and the final concentrations of SaA, SaB, RA, DSS, PA, CA, and SG were 576.0, 824.0, 247.2, 948.0, 230.4, 13.1, and 62.0 μg/ml, respectively. The mixed stock solution (QC sample with high concentration) was diluted by 6 and 12 times to obtain QC samples with middle and low concentration, respectively. The QC samples with three concentration levels were prepared for the method validation.

For accuracy, DHI spiked with high, middle, and low QC samples were analyzed in six replicates. Accuracy was calculated by the formula: (mean obtained concentration-original concentration) / spiked concentration × 100%.

To evaluate intra- and inter-day precision, high, middle, and low QC samples were determined in six replicates on the same day and three consecutive days, respectively. Precision of measurements was evaluated by relative standard deviation (RSD, %).

For repeatability, QC samples with three concentration levels were injected every 12 samples throughout the analytical run. Repeatability of each marker compound was also presented as RSD.

To generate calibration curves, the mixed stock solution was diluted with 10% methanol to obtain standard working solutions with six different concentrations. The calibration curves were generated for each analyte by linear regression analysis of the relationship between concentration (x) and response (y). Linear range and correlation coefficient of each analyte were calculated based on corresponding calibration curves. The limit of detection (LOD) and quantification (LOQ) were the concentrations of each analyte when the ratios of signal to noise (S/N) were about 3 and 10, respectively.

#### Sample Preparation

Danhong injection was mixed with GS, NS, GNS, FI, XI, and DGI in a proportion of 1:6.25 (*n* = 6). The mixing proportion was designed in consideration of a clinical fact that 40 ml of DHI, the most commonly used dose of DHI, was often diluted with 250 ml infusion solution in clinic (Chen et al., 2011). The mixtures were all filtered through 0.22 μm microporous membranes and analyzed immediately by UHPLC-TQ/MS to obtain the original contents of each marker compound. Then the mixtures were exposed at room temperature (25°C) for 2, 4, and 6 h, respectively. The contents of marker compounds at different time points were also analyzed by UHPLC-TQ/MS.

#### Statistical Analysis

The contents of marker compounds in mixtures were expressed as Mean ± SD. The content changes of each marker compound in different mixtures were obtained by the following formula: content of marker compound at different time point / original content of marker compound. In consideration of analytical fact that variation of 5% was acceptable for the error of instrumental response, content change within the range of 0.95–1.05 was defined as stable compatibility in our study.

### Chemical Analysis of Global Compounds by UHPLC-QTOF/MS

Danhong injection is a mixed extraction of two typical Chinese species, the radix and rhizome of *Salvia miltiorrhiza* Bunge (Labiatae) and the dry flower of *Carthamus tinctorius* L. (Asteraceae), which contains many kinds of compounds including phenolic acids, quinochalcones, flavonoid glycosides, iridoid glycosides, organic acids, amino acids, and nucleosides (Zhang et al., 2016). Salvianolic acids are main bioactive compounds in DHI, and can be used as marker compounds for quality control of DHI (Liu H.T. et al., 2013; Liu X. et al., 2013). However, phenolic acids cannot reflect the chemical stability of other components in DHI. Therefore, in addition to phenolic acids, we also want to analyze the contributions of other compounds to the chemical stability when DHI was mixed with different infusion solutions. Here, we developed a semi-quantitative method of simultaneous determination of global compounds (80–1000 Da) in DHI by UHPLC-QTOF/MS, and evaluated the compatible stability according to the response changes of global compounds when DHI was mixed with six infusion solutions (GS, NS, GNS, FI, XI, and DGI) for different time (0, 2, 4, and 6 h) at room temperature (25°C).

#### Liquid Chromatography

The instruments and parameters of chromatographic analysis here were the same as that of UHPLC-TQ/MS. Except for the condition of gradient eluting: 5–5% B (0–1.0 min), 5–40% B (1.0–9.0 min), 40–80% B (9.0–10.0 min), 80–80% B (10.0–12.0 min), 80–5% B (12.0–12.5 min).

#### Mass Spectrometry

Mass spectrometry was performed on Waters Synapt^TM^ QTOF/MS (Waters Corp., Milford, MA, United States) equipped with an ESI source. The conditions used for ESI source were optimized as the following: source temperature 120°C, desolvation temperature 350°C, capillary voltage 3000 V, sample cone voltage 30 V, extraction cone voltage 2 V. All mass data were acquired by using LockSpray^TM^ to ensure mass accuracy and reproducibility. Leucine – Enkephalin was used as the lock mass at a concentration of 200 pg/ml.

#### Sample Preparation

Danhong injection was mixed with GS, NS, GNS, FI, XI, and DGI in a proportion of 1:6.25 (*n* = 6). The mixtures were exposed at room temperature (25°C) for different time (0, 2, 4, and 6 h), and filtered through 0.22 μm microporous membranes before they were analyzed by UHPLC-QTOF/MS.

#### Statistical Analysis

The MS data of all determined samples were analyzed by MassLynx software (version 4.1, Waters Corp. Milford, MA, United States) for peak detection and alignment. For data collection, the method parameters were set as the following: retention time range 0.5–12.0 min, mass range 80–1000 Da, retention time tolerance 0.05 min and mass tolerance 0.01 Da. The resulting data was analyzed by principal component analysis (PCA) with EZinfo 2.0 software. Coordinates of each sample obtained from PCA score plot were used to calculate the deviation distances (Li W. et al., 2014). For each infusion solution, the samples with compatible time of 0 h were set as the reference points (*x*_0_, *y*_0_). The deviation distances between samples with compatible time of 2, 4, and 6 h (*x, y*) to the reference points (*x*_0_, *y*_0_) were calculated according to the following formula: ${\left[{\left(x-x_{0}\right)}^{\wedge 2}+{\left(y-y_{0}\right)}^{\wedge 2}\right]}^{1/2}$. A small deviation distance represented a relatively small change of responses of global components in compatibility of DHI and infusion solutions.

### Identification of Changed Components in Compatibility

Supervised orthogonal partial least squared discriminant analysis (OPLS-DA) was conducted to differentiate the samples with compatible time of 2, 4, or 6 h from that with compatible time of 0 h for each infusion solution. Ions that have the same *t*_R_ (tolerance of 0.05 min) and *m/z* value (tolerance of 0.01 Da) from different samples were considered to be the same ion. The observations that have a large absolute value of *p*(corr) [1] and a large absolute value of the coefficients were displayed in the S-Plot. These are the observations that differentiate the most between the two groups (Guo et al., 2015). That is to say, the responses of these observations are significantly decreased (disappeared) or increased (newly generated) in the compatibility of DHI and infusion solutions.

### Bioactivity Assay of Antioxidant Effect on HUVEC

Danhong injection was widely used for the treatment of various cardiovascular and cerebrovascular diseases, mainly due to its anti-oxidant, anti-inflammatory and anti-thrombosis activities. Among all the bioactivities, anti-oxidant activity is the most important and recognized one (He et al., 2012; Zhao et al., 2012; Zhou et al., 2014; Wang et al., 2016). Therefore, we chose anti-oxidant activity to evaluate the biological stability when DHI was mixed with six infusion solutions (GS, NS, GNS, FI, XI, and DGI) for different time (0, 2, 4, and 6 h) at room temperature (25°C).

#### Cell Culture

Human umbilical vein endothelial cells were seeded at a density of 15,000 cells/well in 96-well plates and cultured at 37°C in a humidified incubator containing 5% CO2. The culture medium for HUVEC was F-12K nutrient mixture supplemented with 10% FBS (v/v), 1% P/S (v/v), 1% ECGS (v/v) and 0.01% heparin (w/v).

#### Antioxidant Activity Assay

Danhong injection was mixed with GS, NS, GNS, FI, XI, and DGI in a proportion of 1:6.25 (n = 6). The mixtures were exposed at room temperature (25°C) for different time (0, 2, 4, and 6 h), and then incubated with HUVEC for 1 h (mixture:medium = 1:10, v/v). Then, HUVEC was treated with 3 mM H_2_O_2_ for 1 h to induce oxidative stress (Safaeian et al., 2016). Vitamin C (2.30, 0.76, 0.25, 0.08, 0.028, 0.0093, and 0.0031 mM) were used as positive controls. The cell viability was evaluated by MTT assay, and absorbance at 570 nm was used to reflect cell viability.

#### Statistical Analysis

The hierarchy of antioxidant capacity of mixtures was presented as vitamin C equivalent antioxidant capability (VCEAC). VCEAC is defined as antioxidant capacity equivalent to the concentration of vitamin C (Kim et al., 2002; Kim and Lee, 2004). The vitamin C standard curve was generated by linear regression analysis of the relationship between concentration (x) and absorbance (y). VCEAC of each mixture was calculated according to the vitamin C standard curve and corresponding absorbance.

## Results

### Stability of DHI

**FIGURE 3:**
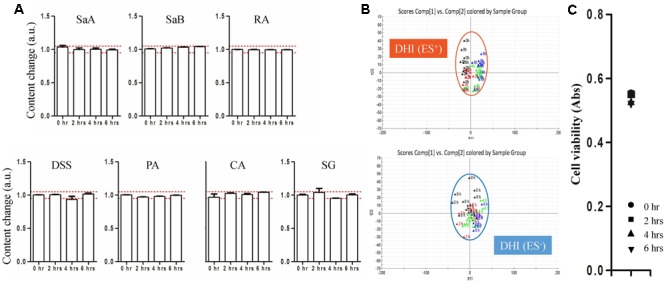
**(A)** Content changes of SaA, SaB, RA, DSS, PA, CA, and SG when DHI was exposed at room temperature (25°C) for different time (0, 2, 4, and 6 h). The samples limited between the red lines were accepted as stable compatibility. **(B)** PCA score plot in positive and negative mode when DHI was exposed at room temperature (25°C) for different time (0, 2, 4, and 6 h). Samples near each other in the plot were probably similar. **(C)** Effect of DHI on oxidative stress induced by H_2_O_2_ when DHI was exposed at room temperature (25°C) for different time (0, 2, 4, and 6 h).

The chemical and biological stability of DHI were evaluated by established strategy before the evaluation of compatible stability of DHI and infusion solutions. For the evaluation of chemical stability of DHI, DHI was diluted by water for injection, a kind of sterile and distilled water used in the production of DHI, in a proportion of 1:6.25 to reach the same content level in the LC/MS analysis of DHI and mixtures. The results showed that the content change of marker compounds was limited in the range of 0.954–1.046 (**Figure 3A**), and the deviation distance of response of global components was limited in the range of 0.27–0.50 (**Figure 3B**), which indicated the chemical stability of DHI when DHI was exposed at room temperature (25°C) for 6 h. Furthermore, the protective effect of DHI on oxidative stress showed no significant changes after the exposure of DHI (**Figure 3C**), which indicated that DHI was biologically stable at room temperature (25°C) within 6 h. Therefore, when DHI was mixed with infusion solutions, if the contents of marker compounds, the responses of global components or antioxidant capability showed significant changes, which must be induced by the compatibility with infusion solutions.

### Evaluation of Compatible Stability by UHPLC-TQ/MS

A rapid and reliable method to simultaneously quantify marker compounds in DHI such as SaA, SaB, RA, DSS, PA, CA, and SG by UHPLC-TQ/MS was established. The accuracy, precision, and repeatability of seven phenolic acids at three concentration levels were listed in **Table 2**. The overall accuracy of SaA, SaB, RA, DSS, PA, CA, and SG was in the range of 96.4–103.4%. The variations of precision and repeatability were all less than 5%. The calibration curves of marker compounds showed good linear relationship over the determination ranges (*r*^2^ > 0.990). LODs and LOQs of SaA, SaB, RA, DSS, PA, CA, and SG were in the range of 2.23–4.87 and 3.35–7.20 μg/ml, respectively (**Table 3**). The results indicated that our method used to quantify SaA, SaB, RA, DSS, PA, CA, and SG is reliable and reproducible.

**Table 2:** Accuracy, precision and repeatability of marker compounds at three concentration levels by UHPLC-TQ/MS.

Analytes	Concentration (μg/ml)	Accuracy (%)	Precision (RSD, %)		Repeatability (RSD, %)
			Intra-day	Inter-day	
SaA	576.0	96.8	3.7	2.6	2.3
	96.0	99.3	4.1	1.3	1.5
	48.0	102.2	3.0	4.2	1.7
SaB	824.0	99.9	2.2	1.1	1.0
	137.3	97.5	3.3	3.4	2.4
	68.7	98.8	1.9	3.6	3.5
RA	247.2	101.1	1.8	1.4	2.8
	41.2	103.2	2.5	2.5	2.3
	20.6	96.7	3.1	1.9	2.4
DSS	948.0	98.4	1.3	2.0	3.6
	158.0	99.8	3.6	2.8	2.7
	79.0	97.4	3.5	3.1	1.5
PA	230.4	99.9	1.7	1.3	4.4
	38.4	102.3	3.1	3.8	1.3
	19.2	96.4	2.9	1.5	3.5
CA	13.1	100.8	1.7	1.2	4.5
	2.2	98.3	3.5	3.3	2.1
	1.1	96.9	2.6	2.7	2.7
SG	62.0	99.3	4.3	3.0	3.2
	10.3	97.5	1.5	2.5	1.1
	5.2	103.4	3.8	1.7	3.0

**Table 3:** Calibration curves, linear ranges, correlation coefficients, LODs and LOQs of marker compounds by UHPLC-TQ/MS.

Analytes	Calibration curve	Linear range (μg/ml)	Correlation coefficient	LOD (μg/ml)	LOQ (μg/ml)
SaA	*y* = 28207*x*-16221	36.0–576.0	0.995	4.80	7.20
SaB	*y* = 1294*x*+94429	50.6–824.0	0.994	2.89	4.34
RA	*y* = 3799*x*-5334	5.15–247.2	0.993	2.23	3.35
DSS	*y* = 704*x* + 23536	52.0–948.0	0.996	4.34	6.51
PA	*y* = 5144*x* + 45051	7.2–230.4	0.998	4.87	7.31
CA	y = 8469x + 4715	0.10–13.1	0.990	0.70	1.05
SG	y = 414x-5330	0.97–62.0	0.997	0.50	0.75

**FIGURE 4:**
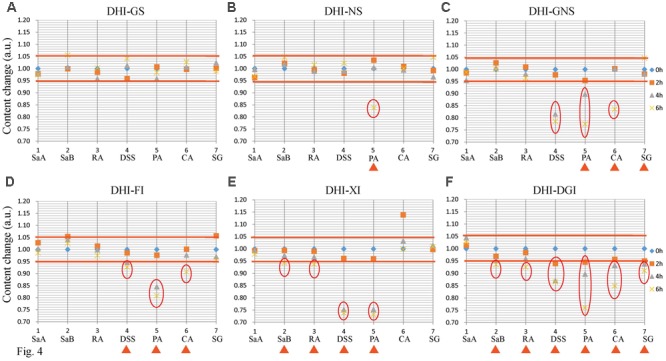
Content changes of SaA, SaB, RA, DSS, PA, CA, and SG when DHI was mixed with **(A)** GS, **(B)** NS, **(C)** GNS, **(D)** FI, **(E)** XI and **(F)** DGI for different time (0, 2, 4, and 6 h) at room temperature (25°C). The samples limited between the red lines were accepted as stable compatibility. Significant changes of marker compounds were marked by triangles and circles.

Among all the six infusion solutions, GS–DHI showed the best stability. No significant changes of marker compounds were observed when DHI was mixed with GS for 6 h at room temperature. However, the compatible stability of DHI–DGI was the worst among all the six infusion solutions. All the marker compounds except SaA decreased significantly when DHI was mixed with DGI. When DHI was mixed with other infusion solutions, different marker compounds would decrease at different time points (**Figure 4**), and the detailed results were listed in **Table 4**. When DHI was mixed with infusion solutions, the mixtures should be used as soon as possible, because the content of marker compounds will decrease with the time during the compatibility.

**Table 4:** Content changes of marker compounds in the compatibility of DHI and infusion solutions.

Infusion solutions	Compatible time	Content changes of marker compounds(%)						
		SaA	SaB	RA	DSS	PA	CA	SG
DHI-GS	–	–	–	–	–	–	–	–
DHI-NS	6 h	–	–	–	–	16.1	–	–
DHI-GNS	4 h	–	–	–	18.4	10.3	–	–
	6 h	–	–	–	21.4	22.6	16.4	–
DHI-FI	4 h	–	–	–	5.3	15.4	–	–
	6 h	–	–	–	7.2	19.2	9.2	–
DHI-XI	4 h	–	–	–	24.8	24.8	–	–
	6 h	–	5.7	6.2	26.1	26.9	–	–
DHI-DGI	2 h	–	–	–	6.0	5.4	–	–
	4 h	–	–	–	12.9	10.3	6.7	6.2
	6 h	–	6.4	7.3	21.4	22.6	15.1	9.0

*– Indicated no significant change. The information of time points with compatible stability (content changes of marker compounds were less than 5%) were not shown in this table.*

### Evaluation of Compatible Stability by UHPLC-QTOF/MS

**FIGURE 5:**
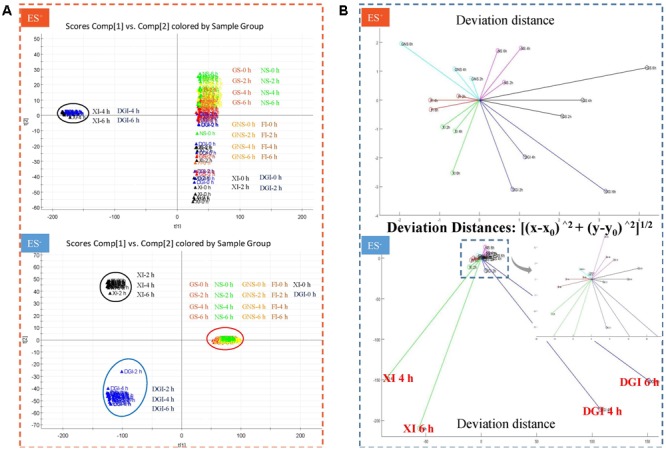
**(A)** Principal component analysis (PCA) score plot in positive and negative mode when DHI was mixed with six infusion solutions (GS, NS, GNS, FI, XI, and DGI) for different time (0, 2, 4, and 6 h) at room temperature (25°C). Each point corresponded to one sample, and samples in different groups were presented in different colors. Samples near each other in the plot were probably similar, and samples far away from each other were different. **(B)** Deviation distance in positive and negative mode when DHI was mixed with six infusion solutions (GS, NS, GNS, FI, XI, and DGI) for different time (0, 2, 4, and 6 h) at room temperature (25°C). A greater distance indicated a greater change in response of global compounds and a worse stability of compatibility.

The UHPLC-QTOF/MS method of data collection we set (retention time range 0.5–12.0 min, mass range 80–1000 Da) could detect most of the components in DHI. Responses of all the compounds were weighted to obtain indices in PCA. Score *t*[1] and *t*[2] are the most important indices summarizing and separating the dataset. Hence, the PCA score plot of *t*[1] versus *t*[2] give a picture of our dataset. Samples near each other in the plot are similar, and samples far away from each other are different. In our assay, DHI was mixed with six infusion solutions, each solution had four time points, and each time point had five replicate samples. Therefore, there were a total of 120 points in the PCA score plot. Each point corresponded to one sample, and samples in different groups were presented in different colors (**Figure 5A**).

When DHI was mixed with different infusion solutions, those compounds with positive response were much more stable than those with negative response, as the deviation distances in positive mode are much smaller than that in negative mode. When DHI was mixed with XI or DGI, the compatible stability was the worst among all the infusion solutions, because their deviation distances showed significant changes in both positive and negative modes (**Figure 5B**). When DHI was mixed with infusion solutions, the mixtures should be used as soon as possible, because the response of global components will be getting worse with the time during the compatibility.

### Identification of Changed Components in Compatibility

**FIGURE 6:**
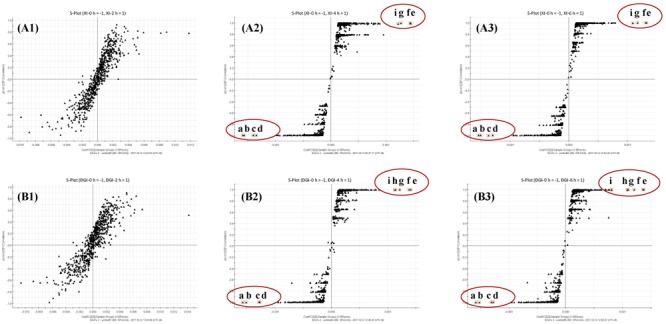
**(A)** S-plots from OPLS-DA dataset of compatibility of DHI and XI at different time points (**A1**, 2 h; **A2**, 4 h and **A3**, 6 h); **(B)** S-plots from OPLS-DA dataset of compatibility of DHI and DGI at different time points (**B1**, 2 h; **B2**, 4 h and **B3**, 6 h). Observations marked with a–d were components that disappeared in the compatibility, and that marked with e–i were the components that newly generated in the compatibility.

Markers that contribute to the group separation can be clearly displayed as the dots in the S-plot. When DHI was mixed with infusion solutions, those increased compounds were located in the first quadrant, while the decreased compounds were located in the third quadrant. The compound located in the margin region of the first and third quadrant (**Figure 6**, a–i) were the markers that can differentiate the two groups. And the more compounds that located in the margin region of the quadrant, the greater change in the global components of DHI. In the compatibility of DHI and XI or DGI, the chemical composition of DHI showed no significant changes after 2 h of compatibility (**Figures 6A1,B1**). However, after 4 and 6 h of compatibility, the changed components were significantly increased (**Figures 6A2,A3,B2,B3**). Notably, the decreased (a–d) or increased (e–i) components were consistent in the samples with compatible time of 4 h and that of 6 h for each infusion solution. Furthermore, the changed components in the compatibility of DHI and XI, and that of DHI and DGI were also consistent (**Table 5**), which indicated that the main unstable components and the chemical reactions in compatibility of DHI and infusion solutions were probably the same.

**Table 5:** Disappeared and newly generated products in the compatibility of DHI and two instable infusion solutions (XI and DGI).

	DHI-XI (4 h)			DHI-XI (6 h)	
	*t*_R_	*m/z*		*t*_R_	*m/z*
**Disappeared products**					
a	4.73	137.0235	a	4.73	137.0233
b	7.65	161.0229	b	7.65	161.0299
c	8.12	519.0897	c	8.12	519.0895
d	7.65	359.0744	d	7.65	359.0744
**Newly generated products**					
e	4.30	137.0236	e	7.30	161.0229
f	7.31	161.0299	f	4.26	137.0236
g*	7.30	357.0743	g*	7.30	357.0738
h	7.82	519.0901	h	7.81	519.0896

	**DHI-DGI (4 h)**			**DHI-DGI (6 h)**	
	***t*_R_**	***m/z***		***t*_R_**	***m/z***

**Disappeared products**					
a	4.72	137.0233	a	4.72	137.0233
b	7.65	161.0230	b	7.65	161.0230
c	8.11	519.0905	c	8.11	519.0904
d	7.65	359.0741	d	7.65	359.0744
**Newly generated products**					
e	4.28	137.0235	f	7.29	161.0229
f	7.29	161.0231	e	4.28	137.0233
g^∗^	7.30	357.0743	g*	7.30	357.0736
h	7.76	519.0900	h	7.76	519.0887
i	7.81	519.0906	i	7.81	519.0903

*The structure of marked product ^∗^ was identified according to the degradation regularity of salvianolic acids. The information of time point with stable compatibility (2 h) was not listed in this table.*

**FIGURE 7:**
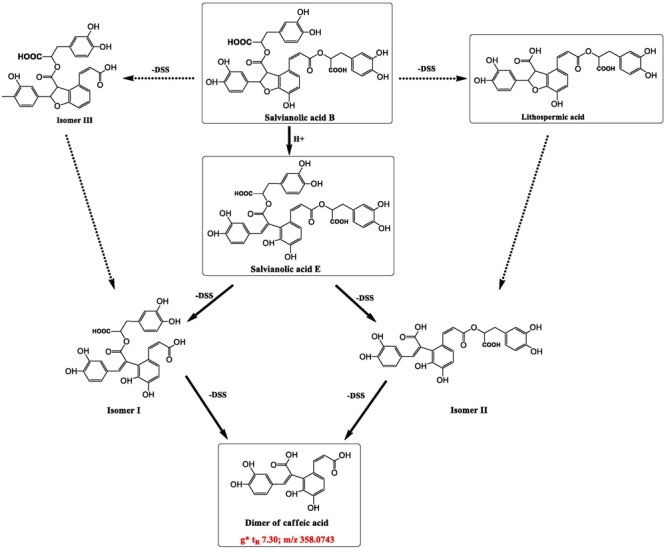
Speculated structure of a newly generated compound in compatibility of DHI and XI or DGI that marked as *g* (*t*_R_ 7.30, *m/z* 357.0743), and possible way of formation of compounds *g* from some salvianolic acids in DHI.

At the bottom of the third quadrant, a–d were those components disappeared after DHI was mixed with infusion solution; and at the top of the first quadrant, e–i were newly generated components in the compatibility of DHI and XI or DGI (**Figure 6**). According to the precise molecular weight of these changed components, and the degradation pathway of salvianolic acids in DHI, we tried to speculate the structure of these newly generated components. Finally, we speculated that the newly generated compound *g* (*t*_R_ 7.30, *m/z* 357.0743) might be the dimer of caffeic acid (**Figure 7**). As shown in **Figure 7**, when DHI was mixed with some injection solutions, salvianolic acid B, salvianolic acid E, lithospermic acid, and some other salvianolic acids in DHI was transformed to other components by losing one or two molecules of Danshensu, and therefore generated compound *g* (Zhou et al., 2010; Zheng and Qu, 2011). However, the components in DHI are complicated, and the reactions in the compatibility are much more complex than we expected, thus additional attention should be paid to the research of the structure of other unknown components in the compatibility in future.

### Evaluation of Compatible Stability Based on Bioactivity Assay

Treatment of H_2_O_2_ (3 mM, 1 h) induced oxidative stress on HUVEC with a decreased viability of 55.1%. Vitamin C could protect HUVEC from H_2_O_2_ induced oxidative stress in a concentration dependent manner. At the concentrations range from 0.084 to 2.30 mM, vitamin C showed a good dose–effect relationship and the standard curve was obtained as following: y = 0.119x + 0.454 (*r*^2^= 0.995).

When DHI was mixed with different infusion solutions, the antioxidant capability of DHI decreased with time. However, the differences among infusion solutions were not significant according to the cell viability; the cell viability was limited in the range of 0.50–0.61. Therefore, VCEAC, an indicator with better sensitivity, was adopted to magnify the differences to reflect the hierarchy of antioxidant capability (Kim et al., 2002; Kim and Lee, 2004). As shown in **Table 6**, VCEAC were sensitive enough to present the differences of antioxidant capability of different mixtures. When DHI was mixed with GS, the antioxidant capability was the best among all the tested solutions (VCEAC = 0.83–1.31 mM). However, the antioxidant capability of DHI-XI mixture was the worst among all the infusion solutions, the antioxidant capability (VCEAC = 0.46–1.03 mM) decreased by 51.2% after 6 h. When DHI was mixed with infusion solutions, the mixtures should be used as soon as possible, because the antioxidant capability of DHI decreased with the time during the compatibility.

**Table 6:** Antioxidant capacity equivalent to the concentration of vitamin C (VCEAC) when DHI was mixed with GS, NS, GNS, FI, XI, and DGI (Mean ± SD).

Compatible time (h)	DHI-GS	DHI-NS	DHI-GNS	DHI-FI	DHI-XI	DHI-DGI
0	1.31 ± 0.04	1.11 ± 0.07	0.82 ± 0.05	0.97 ± 0.02	1.03 ± 0.08	1.11 ± 0.03
2	1.24 ± 0.02	0.92 ± 0.30	0.77 ± 0.01	0.96 ± 0.00	0.89 ± 0.08	1.09 ± 0.05
4	1.06 ± 0.03	0.65 ± 0.03	0.76 ± 0.03	0.92 ± 0.05	0.64 ± 0.03	0.98 ± 0.10
6	0.84 ± 0.08	0.55 ± 0.04	0.40 ± 0.05	0.89 ± 0.09	0.46 ± 0.05	0.79 ± 0.03

## Discussion

Multicomponent is the characteristic of TCMI, therefore, when evaluating TCMI stability, several marker compounds in TCMI are not enough to represent global components or biological activities of TCMI. Information obtained from any single method to evaluate the compatible stability of DHI and infusion solutions is limited, therefore different methods should be combined to make a comprehensive conclusion. In this paper, we established a comprehensive strategy composed of three different methods to evaluate the chemical and biological stability of DHI.

First, we have developed a rapid and reliable method to evaluate the chemical stability of seven marker components (SaA, SaB, RA, DSS, PA, CA, and SG) in DHI by UHPLC-TQ/MS. This method can be applied to other TCMIs such as Danshen injection that contain these phenolic acids. Second, we have established a novel method to assess the chemical stability of global components with molecular weight less than 1000 Da in DHI by UHPLC-QTOF/MS. Moreover, the response changes of global components were processed by PCA, and presented as deviation distance. This quantitative method can be used to evaluate the response changes of global components in DHI. Third, we have successfully established a cell based bioactivity assay to evaluate the antioxidant effect of DHI. Moreover, VCEAC was applied to quantify and magnify the differences of antioxidant capability when DHI was mixed with different infusion solutions. This biological assay can be applied to evaluate the antioxidant effect of other TCMIs.

According to the results of chemical analysis of marker compounds, GS was the best infusion solution for DHI, and DGI was the worst one. Based on the results of chemical analysis of global components, the worst infusion solutions for DHI were XI and DGI. The compatible stability of DHI and other infusion solutions were much better, and there was no significant difference between these infusion solutions, thus we could not propose the best one. According to the results of bioactivity assay, we can determine that GS was the best infusion solution for DHI, and XI was the worst one. In conclusion, as evaluated by the established comprehensive strategy, GS was the best infusion solution for DHI, XI and DGI were the worst infusion solutions for DHI (**Figure 1**). Furthermore, we can conclude that the mixtures of DHI and infusion solutions should be used as soon as possible, because the marker compounds, global components and antioxidant capability would decrease over time during the compatibility.

Our results showed that XI and DGI were the worst infusion solutions for DHI. And UPLC-QTOF/UPLC-TQ analysis showed that when DHI was mixed with XI or DGI, salvianolic acids in DHI were not stable and should be degraded resulting in the reduction of original composition and generation of new components. In order to explain why XI and DGI lead to the instability of DHI, we have compared the physiochemical properties of XI, DGI and other infusion solutions. DGI is a colloidal injection containing dextran 40, a kind of macromolecular compound (MW 32,000–42,000) and the polymer of glucose, which is different with other infusion solutions. The other physicochemical properties such as pH value (3.5–6.5) and osmotic pressure (280 mOsmol/kg) are similar to those of other infusion solutions. Therefore, the special dispersion system of DGI might induce the change of salvianolic acids in DHI.

As for XI, its physicochemical properties (molecular weight of 152.12, pH value of 4.5–7.0 and osmotic pressure of 329 mOsmol/kg) is similar with other infusion solutions. So the reasons why XI induce the instability of DHI may not be explained by its physicochemical properties. Although the chemical changes of DHI are similar when mixed with XI and DGI, the reasons and mechanisms is different and need further exploration.

As reported previously, deviation distance of different samples can be calculated from the responses of endogenous small molecule compounds based on metabolomics and multivariate statistical analysis (PCA, PLS-DA and so on) and can be used to quantitatively evaluate the efficacy of Chinese medicine (Li W. et al., 2014). In PCA or PLS-DA score plot, the deviation distance between different samples represent the similarity of global response of endogenous small molecule compounds, and thus reflect the similarity of these samples. If the deviation distance between two groups come to closer, which means that the global response of endogenous small molecule compounds in these two groups become more similar, and indicate a close relationship between these two groups. Based on the above strategy, we applied PCA and deviation distance to evaluate the similarity between DHI samples mixed with infusion solutions and therefore reflect their stability.

## Conclusion

In this paper, we established a comprehensive strategy composed of three different methods to evaluate the chemical and biological stability of DHI. We applied this strategy to assess the compatible stability of DHI and six infusion solutions (GS, NS, GNS, FI, XI, and DGI), which were commonly used in combination with DHI in clinic. As evaluated by the established comprehensive strategy, GS was the best infusion solution, XI and DGI were the worst infusion solutions for DHI. In the compatibility of DHI and XI or DGI, salvianolic acids in DHI would be degraded, resulting in the changes of biological activities. Our study provided references for choosing the reasonable infusion solutions for DHI, which could contribute the improvement of safety and efficacy of DHI. Moreover, the established strategy may be applied for the compatible stability evaluation of other TCMIs.

## Author Contributions

J-PL, J-MG, B-CZ, Z-ST, and J-AD designed the study. J-PL, YL, E-XS, Z-HZ, and KZ conducted the experiments. J-PL, J-MG, J-AD, and Y-PT wrote and revised the manuscript. All authors approved the final version to be published.

## Conflict of Interest Statement

The authors declare that the research was conducted in the absence of any commercial or financial relationships that could be construed as a potential conflict of interest.

## References

[B1] Binnor A. K. Mukkanti K. Suryanarayana M. V. Roy S. B. (2013). Stability-indicating UPLC method for tramadol HCl impurities in the tramadol injection after dilution by infusion fluids (5% Dextrose and 0.9% Sodium Chloride). *Sci Pharm.* 81 1003–1015. 10.3797/scipharm.1305-20 24482769 PMC3867236

[B2] Chen F. C. Shi X. Y. Li P. Yang J. G. Zhou B. H. (2015a). Stability of butorphanol-tropisetron mixtures in 0.9% sodium chloride injection for patient-controlled analgesia use. *Medicine (Baltimore)* 94:e432. 10.1097/md.0000000000000432 25674732 PMC4602760

[B3] Chen F. C. Xiong H. Liu H. M. Fang B. X. Li P. (2015b). Compatibility of butorphanol with granisetron in 0.9% sodium chloride injection packaged in glass bottles or polyolefin bags. *Am. J. Health Syst. Pharm.* 72 1374–1378. 10.2146/ajhp140824 26246294

[B4] Chen Q. Yi D. Xie Y. Yang W. Yang W. Zhuang Y. (2011). Analysis of clinical use of Danhong injection based on hospital information system. *Zhongguo Zhong Yao Za Zhi* 36 2817–2820. 10.4268/cjcmm20112016 22292374

[B5] Guo J. Lu Y. Shang E. Li T. Liu Y. Duan J. (2015). Metabolite identification strategy of non-targeted metabolomics and its application for the identification of components in Chinese multicomponent medicine Abelmoschus Manihot L. *Phytomedicine* 5 579–587. 10.1016/j.phymed.2015.02.002 25981925

[B6] He Y. Wan H. Du Y. Bie X. Zhao T. Fu W. (2012). Protective effect of Danhong injection on cerebral ischemia-reperfusion injury in rats. *J. Ethnopharmacol.* 144 387–394. 10.1016/j.jep.2012.09.025 23010366

[B7] Hu Z. Wang H. Fan G. Zhang H. Wang X. Mao J. (2016). Effect of danhong injection on the mobilisation of endothelial progenitor cells to vascular repair after percutaneous coronary intervention: a randomised controlled trial. *Lancet* 388(Suppl. 1), S34 10.1016/s0140-6736(16)31961-4

[B8] Kim D. O. Lee C. Y. (2004). Comprehensive study on vitamin C equivalent antioxidant capacity (VCEAC) of various polyphenolics in scavenging a free radical and its structural relationship. *Crit. Rev. Food Sci. Nutr.* 44 253–273. 10.1080/10408690490464960 15462129

[B9] Kim D. O. Lee K. W. Lee H. J. Lee C. Y. (2002). Vitamin C equivalent antioxidant capacity (VCEAC) of phenolic phytochemicals. *J. Agric. Food Chem.* 50 3713–3717. 10.1021/jf020071c 12059148

[B10] Li J. Guo J. Duan J. Fan X. Du X. Sun J. (2014). Literature study of drug combination type and incompatibility of traditional Chinese medicine injections. *Chin. J. Drug Control* 11 432–438.

[B11] Li W. Tang Y. Guo J. Shang E. Qian Y. Wang L. (2014). Comparative metabolomics analysis on hematopoietic functions of herb pair Gui-Xiong by ultra-high-performance liquid chromatography coupled to quadrupole time-of-flight mass spectrometry and pattern recognition approach. *J. Chromatogr. A* 1346 49–56. 10.1016/j.chroma.2014.04.042 24794940

[B12] Liu H. T. Wang Y. F. Olaleye O. Zhu Y. Gao X. M. Kang L. Y. (2013). Characterization of in vivo antioxidant constituents and dual-standard quality assessment of Danhong injection. *Biomed. Chromatogr.* 27 655–663. 10.1002/bmc.2842 23233112

[B13] Liu X. Wu Z. Yang K. Ding H. Wu Y. (2013). Quantitative analysis combined with chromatographic fingerprint for comprehensive evaluation of Danhong injection using HPLC-DAD. *J. Pharm. Biomed. Anal.* 76 70–74. 10.1016/j.jpba.2012.12.013 23298908

[B14] Myers A. L. Zhang Y. Kawedia J. D. Shank B. R. Deaver M. A. Kramer M. A. (2016). Stability of tacrolimus injection diluted in 0.9% sodium chloride injection and stored in Excel bags. *Am. J. Health Syst. Pharm.* 73 2083–2088. 10.2146/ajhp150677 27919876

[B15] Safaeian L. Sajjadi S. E. Javanmard S. H. Montazeri H. Samani F. (2016). Protective effect of Melissa officinalis extract against H2O2-induced oxidative stress in human vascular endothelial cells. *Res. Pharm. Sci.* 11 383–389. 10.4103/1735-5362.192488 27920820 PMC5122827

[B16] Wang Y. Jiang Z. Yang F. Chai X. Zhu Y. Zhao X. (2016). Establishment of a ternary network system for evaluating the antioxidant fraction of Danhong injection. *Biomed. Chromatogr.* 30 1666–1675. 10.1002/bmc.3739 27062150

[B17] Zhang Q. Q. Dong X. Liu X. G. Gao W. Li P. Yang H. (2016). Rapid separation and identification of multiple constituents in Danhong Injection by ultra-high performance liquid chromatography coupled to electrospray ionization quadrupole time-of-flight tandem mass spectrometry. *Chin. J. Nat. Med.* 14 147–160. 10.1016/s1875-5364(16)60008-0 26968681

[B18] Zhao T. Zhao B. Wu H. Zhao M. (2012). Research progress on the protection of vascular endothelial injury by Danhong injection. *China Med. Herald* 9 31–35.

[B19] Zheng X. Qu H. (2011). Characterisation of the degradation of salvianolic acid B using an online spectroscopic analysis system and multivariate curve resolution. *Phytochem. Anal.* 23 103–109. 10.1002/pca.1330 21692119

[B20] Zhou L. Zhang X. Xu W. Ma X. Jia Z. Zheng Y. (2010). Studies on the stability of salvianolic acid B as potential drug material. *Phytochem. Anal.* 22 378–384. 10.1002/pca.1291 21226127

[B21] Zhou P. He Y. Yang J. H. Zhang Y. Y. Zhou H. F. Zhao T. (2014). Protective mechanism of Danhong injection on brain microvascular endothelial cells injured by hypoxic. *Zhongguo Zhong Yao Za Zhi* 39 4844–4848. 10.4268/cjcmm20142428 25898589

